# Revealing the comprehensive genomic landscapes of multidrug-resistant *Enterobacter hormaechei* isolates from cutaneous and soft tissue infections in hospitals of Kolkata and Malda, West Bengal, India

**DOI:** 10.1128/mra.00187-25

**Published:** 2025-07-02

**Authors:** Indrani Sarkar, Soma Sarkar, Debarati Chattopadhay, Uttam Kumar Nath, Surojit Das, Saugata Hazra

**Affiliations:** 1 Department of Biosciences and Bioengineering, Indian Institute of Technology - Roorkee 729296, Roorkee, Uttarakhand, India; 2 Department of Medical Oncology and Haematology, All India Institute of Medical Sciences 442339, Rishikesh, Uttarakhand, India; 3 Microbiology, Infectious Diseases & Beleghata General Hospital, Kolkata, West Bengal, India; 4 Biomedical Laboratory Science and Management, Vidyasagar University 75000, Midnapore, West Bengal, India; 5 Centre for Nanotechnology, Indian Institute of Technology - Roorkee 30112 , Roorkee, Uttarakhand, India; Rochester Institute of Technology, Rochester, New York, USA

**Keywords:** antimicrobial resistance, *Enterobacter*, wound, pus, clinical samples, *de novo* genome sequencing

## Abstract

Here, we report five *Enterobacter hormaechei* isolated from pus and wound samples of skin and soft tissue infected patients from Kolkata and Malda, West Bengal, India. They were found to contain potent antimicrobial resistance (AMR) genes in their chromosome and predicted plasmids, which has implications for their clinical impacts on public health.

## ANNOUNCEMENT

The genus *Enterobacter* (family—Enterobacteriaceae) is generally composed of Gram-negative rods and can be isolated from a range of habitats, i.e., from soil to human (1). Many are nosocomial pathogens with multiple antimicrobial resistance (AMR) genes (2). Here, we report the *de novo* whole-genome sequences of five clinical *Enterobacter hormaechei* strains, among which three were isolated from wounds (from Kolkata, India) and two were isolated from abscesses (from Malda) (IEC no. VU/IEC/122/2020, Vidyasagar University, India). The isolates were recovered from inpatients admitted to tertiary-care multispecialty hospitals in West Bengal, India and were identified using the automated VITEK2 system (bioMérieux SA, Marcy l’Etoile, France) (3). The isolates were revived from the glycerol-preserved bacterial repository onto Luria-Bertani agar (HiMedia, India) for whole-genome sequencing. The genomic DNA was extracted with the Xploregen Bacterial DNA extraction kit following the standard protocol, and the Illumina NovaSeq 6000 platform was used for whole-genome sequencing (4). Library preparation was done through the Kapa DNA library kit. It was a paired-end sequencing with 151 bp read length. Quality control was done using FastQC and MultiQC. The *de novo* primary and secondary assembly was performed using Unicycler (version 2024) and MeduSa (version 2024). BUSCO 5.8.2 (5) was used to calculate the completeness of the genomes. Prokka 1.14.5 (6) was used for gene annotation. ResFinder 4.6.0 (7) and CARD databases 4.0.0 (8) were used for the prediction of AMR genes. Plasmid Finder 2.1 (9) was exploited for the prediction of plasmid sequences from the secondary assembly data. Default parameters were used for all the software and web servers.

The genome sequencing resulted in 7.2, 7.8, and 6.2 M reads for ECC_004, ECC_006, and ECC_007, respectively, and 8.8 M reads for both ECC_011 and ECC_012. The average GC was 55% (Table 1). The chromosomes of these five isolates contained aac(3), aac(7) genes, giving them resistance against gentamicin, tobramycin, amikacin, dibekacin, netilmicin, sisomicin, and ciprofloxacin antibiotics. Among beta-lactamases (*bla*), *bla*_OXA_ was present in all five genomes. The *bla*_NDM_ was present in all except ECC_011, *bla*_ACT_ was present in all except ECC_006, and *bla*_CTX-M_ and *bla*_TEM_ were present in all except ECC_007. Interestingly, *bla*_TEM_ and *bla*_OXA_ were predicted in the IncFII_pMET plasmid in ECC_004. ECC_006, ECC_007, ECC_011, and ECC_012 contained col(pHAD28) plasmid which possesses *qnrB19*, giving them resistance against ciprofloxacin. ECC_006 contained IncR plasmid with *bla*_LAP_ and *bla*_SHV_. The presence of versatile AMR genes located in the chromosome or plasmid predicts that these isolates are co-resistant to anti-infective treatment regimens, such as third-generation cephalosporins, carbapenems, aminoglycosides, and fluoroquinolones.

**TABLE 1:** Summary of the five *Enterobacter hormaechei* isolates

Isolate number	Specimen	Genome completeness (%)	Genome coverage	Size of the secondary assembly	GC %	No. of contigs	No. of genes (total)	No. of CDS^*a*^	No. of genes (coding)
ECC_004	Wound	98.9	217.69×	5,000,420	54	20	4,452	4,448	4,410
ECC_006	Abscess	99.1	222.97×	5,314,759	55	23	4,537	4,534	4,493
ECC_007	Abscess	98.9	196.61×	4,806,680	56	8	4,204	4,201	4,163
ECC_011	Wound	98.6	273.3×	4,883,958	55	29	4,329	4,326	4,278
ECC_012	Wound	98.9	263.24×	5,100,866	55	28	4,567	4,563	4,529

^*a*^ CDS, coding sequences.

## Data Availability

Raw fastq files are available under the BioProject accession number PRJNA1218013 and BioSample IDs SAMN46508903 (ECC_004), SAMN46508904 (ECC_006), SAMN46508905 (ECC_007), SAMN46508908 (ECC_011), and SAMN46508909 (ECC_012). The GenBank accession numbers for the genomes of these five isolates are JBLQSU000000000 (ECC_004), JBLQST000000000 (ECC_006), JBLQSS000000000 (ECC_007), JBLQSP000000000 (ECC_011), and JBLQSO000000000 (ECC_012).
